# Time required to implement a computerized patient decision aid for lupus in outpatient visits

**DOI:** 10.1186/s43058-025-00727-8

**Published:** 2025-04-10

**Authors:** Nathan W. Carroll, Aizhan Karabukayeva, Larry R. Hearld, Diane Kamen, Alfred H. J. Kim, Sonali Narain, Narender Annapureddy, Zineb Aouhab, Maureen McMahon, Vikas Majithia, Cathy Lee Ching, Winn Chatham, Jasvinder A. Singh

**Affiliations:** 1 Department of Health Administration, College of Health Professions, Virginia Commonwealth University, Richmond, VA USA; 2 Hudson College of Public Health, University of Oklahoma Health Sciences, Oklahoma City, OK USA; 3 Department of Health Services Administration, School of Health Professions, University of Alabama at Birmingham, Birmingham, AL USA; 4 Medical University of South Carolina (MUSC), Charleston, SC USA; 5 Washington University School of Medicine, St. Louis, MO USA; 6 Northwell Hospital, Long Island, NY USA; 7 Vanderbilt University, Nashville, TN USA; 8 Loyola University, Chicago, IL USA; 9 University of California at los Angeles (UCLA), Los Angeles, CA USA; 10 University of Mississippi Medical Center, Jackson, MS USA; 11 University of Nevada, Las Vegas, NV USA; 12 Baylor College of Medicine, 7200 Cambridge Street, Houston, TX 77030 USA

**Keywords:** Doctor-patient interaction, Medical decision making, Patient centered care, Patient participation, Decision aid, Shared decision-making, Systemic lupus erythematosus

## Abstract

**Background:**

Patient decision aids have the potential to lower decision conflict for patients and to improve patient-physician communication. However, uptake of decision aids has been poor, in part because the time required to incorporate these into clinical practice is not well understood.

**Objective:**

To estimate the time required for a rheumatology clinic to implement a validated decision aid for patients with lupus.

**Methods:**

Using a cohort of eight implementation sites, study investigators identified the activities required to administer a decision aid. Site coordinators embedded within the clinics timed the duration of each activity. To estimate the effect of viewing the decision aid on the length of the physician–patient interaction, patient visits were timed and the length of visits for patients who viewed the decision aid were compared with visit lengths for three groups of control patients.

**Results:**

Estimates of the effect of the decision aid on patient visit lengths ranged from a reduction of 3 min per visit to an increase of 3.88 min per visit, with five out of six estimates suggesting the decision aid is associated with shorter patient visits. Introducing the decision aid to patients took a mean of 4.12 min (median of 2 min). Identifying patients eligible for the decision aid was a weekly or bi-weekly process for most clinics and took an average of 41.43 min.

**Conclusion:**

The time required for a rheumatology clinic to implement the decision aid for patients with lupus is low. Our results raise questions about why decision aid take up is low among clinical practices, given the benefits the lupus decision aid offers to patients (reduced decisional conflict and better-informed choice of immunosuppressive medications used for the treatment of lupus kidney disease). More research is needed to identify barriers to decision aid adoption.

Contributions to the literature

- Patient decision aids have been shown to improve quality of the decision-making process, lower decision conflict, and improve patient-physician communication. However, adoption of patient decision aids has not been widespread.
- The time required for clinics to incorporate the decision aid into practice is minimal. Moreover, the lengthiest activities required for adoption are conducted by non-physician clinic personnel while decision aid appears to have little if any impact physicians’ workflow
- Inaccurate perceptions of the time required for decision aid implementation, or other implementation barriers, may be important factors inhibiting widespread patient decision aid implementation.

## Background

Despite evidence indicating patient decision aids (PtDA) improve quality of the decision-making process, lower decision conflict and improve patient-physician communication, there has been low uptake outside research settings [1]. One reason may be the real or perceived time required for a physician practice to incorporate a PtDA into its existing processes creates a barrier to adoption. Many decision aid implementation studies do not include information on the time required for implementation.

In this study, we examined the time required to implement a PtDA for people with systemic lupus erythematosus (SLE-referred to as lupus from here onwards), using an observational implementation trial [2]. The study focuses on the time required for physicians and clinic staff to administer the PtDA, since these are the parties ultimately responsible for the adoption decision.

The lupus PtDA was developed based on extensive patient and stakeholder involvement [3–5] and rigorous evidence synthesis [6–8]. In a randomized trial, this lupus PtDA was more effective than the existing American College of Rheumatology (ACR) lupus pamphlet in decreasing decisional conflict and facilitating informed choice for the immunosuppressive medications used for the treatment of lupus kidney disease and other serious organ disease due to lupus [9]. The PtDA was developed and updated based on the International Patient Decision-Aid Standards (IPDAS) [10]. Over a five-year period, from 2019–2024, the PtDA has been used during regular outpatient visits across 15 geographically diverse rheumatology clinics [2]. To date, the PtDA has been viewed by roughly 1,900 patients with lupus. Initially administered on a tablet in-person only, the PtDA has been available as a weblink for telemedicine visits and then as a smartphone app in the last 3 years of implementation. Our study assessed the time required to implement the lupus PtDA in eight of these 15 rheumatology clinics. In the observational implementation trial on which this research is based, PtDA implementation was guided by the Consolidated Framework for Implementation Research (CFIR) [11] and Powell’s typology of implementation strategies [12]. Our focus in this paper is the time component of what the Gold et al. framework terms “intervention costs” [13]. In particular, we focus on time burden from the perspective of an implementing provider (rather than from a patient perspective) so that our results can inform practices concerned about the time required to implement the PtDA. We conceptualize the processes required for a clinic to begin using the PtDA using a time-driven activity-based costing (TDABC) approach. This approach has been used in prior cost of implementation studies [14, 15]. In this article, we present the time results from our TDABC analyses. Cost of implementation estimates, based on the results presented here, are available from the authors upon request.

## Methods

Our data come from eight geographically diverse rheumatology practices. Each practice was part of a larger implementation trial, and the included practices agreed to participate in additional data collection efforts required for this study. Since most of our analyses are quantitative, we follow STROBE reporting guidelines.

To estimate the time associated with implementing the PtDA, we identified the steps required to implement through a series of focus groups with site-coordinators and study personnel. Through these focus groups, we learned that the main activities involved in delivering the PtDA to patients included:

- Identifying which of the scheduled patients are eligible to receive the PtDA
- Introducing the patient to the PtDA and explaining its purpose
- Incorporating the PtDA into the patient/physician interaction

Study coordinators at each site collected data on the time required for PtDA implementation. To begin, study coordinators at each site were provided with training materials that outlined instructions for timing each of the steps required for implementation. Study coordinators at each site used a web-based application to record the time they spent on each step and to communicate that information to study investigators. Study coordinators were able to measure the time required for the first and second steps (identifying eligible patients and introducing the PtDA) directly. Study coordinators typically introduced the PtDA to patients at the same time they were obtaining consent for the study. Since clinics who implement the PtDA in practice (rather than as part of an academic study) would not need to seek informed consent, we asked study coordinators to only time the portions of their patient conversations that related to explaining the PtDA, and not those portions that involved the informed consent process. Similarly, these time estimates only include the time required to introduce the PtDA to patients, and not the full time required for patients to complete the PtDA, since the PtDA is designed to be “handed off” to patients and administration does not require continuous oversight by clinic staff.

The time required for identifying eligible patients and administering the PtDA was also measured by study coordinators. However, the impact of the PtDA on the length of the patient/physician interaction had to be estimated. The time effect of the PtDA on the patient/physician interaction was estimated by comparing the duration of two different patient visits. We constructed this comparison in three different ways. First, we made within-patient comparisons. For this estimate we compared the duration of a patient’s first clinic visit while part of the study (not necessarily the patient’s first visit to the clinic), to the duration of the same patient’s subsequent visit. As a part of the study protocol, the patient was required to review the PtDA as preparation for this first visit, but did not review the aid again prior to their next visit. By comparing visit times for the same patient, we controlled for time-invariant patient characteristics, for instance, the degree to which a patient prefers to make small talk during a visit. The strategy did, however, require us to assume that if viewing the PtDA changed the duration of a patient’s initial visit, this change would not persist during subsequent visits. We believe this is a reasonable assumption given the time between the two visits, which ranged from roughly 2 months to one year. We conducted paired t-tests to compare the mean time required for each visit type, and Wilcoxon signed-rank test to compare median times.

During data collection, we noticed that occasionally the study coordinator would be unable to capture the duration of a patient’s subsequent visit because of challenges identifying when the patient returned for a follow-up visit, site-coordinator turnover, or a variety of other reasons. As a result, our sample contained more initial visit observations than follow-up visit observations, and relying on paired patient visits required discarding unpaired observations. Our second estimation strategy used all the available data and compared the average length of all observed initial visits to the average length of the follow-up visits. Finally, our third estimation strategy compared the average duration of initial visits (before which the patient had viewed the PtDA) to the visit duration for other lupus patients, who were not participating in the study, and hence had not viewed the PtDA. We compared means for each visit type using two-sample t-tests. Medians were compared using Mann–Whitney tests.

## Results

Time data were provided by eight different rheumatology clinic sites. Some basic structural characteristics of these clinics are provided in Table 1. Table 2 reports the number of observations by activity and site. For all activities, the majority of observations came from sites with specialty lupus clinics (as opposed to lupus patients seen in general rheumatology clinics). Most data related to in-person visits, with only nine timed observations related to telehealth visits. As a result, the rest of our results focus on the time required to administer the PtDA in a clinic conducting in-person visits. We analyze time requirements for physicians separately from time required from other clinic personnel.

**Table 1:** Characteristics of participating clinic sites

Characteristic	General rheumatology	Specialty Lupus Clinic
Number of practices	4	4
*Practice type*		
Academic	2	3
Hybrid	0	1
Private	2	0
*Location*		
Urban	2	4
Suburban	2	0
*Clinic size and volume*		
Average number of providers	17	
Mean annual patient volume/clinic (all patients)	10,338	
Mean annual patient volume/clinic (lupus)	632	
Mean annual number of patient visits per year/clinic	22,790	
Mean annual number of in-person visits/clinic	20,700	
Mean annual number of virtual visits/clinic	2,090

**Table 2:** Number of observations and times associated with implementation activities

	**General Rheumatology**		**Specialty Lupus Clinics**	
**Implementation activity**	**Number of observations**	**Minutes of time required (mean/sd)**	**Number of observations**	**Minutes of time required (mean/sd)**
Identify eligible patients	38	21.83 (25.26)	56	54.73 (39.16)
Administer PtDA	47	6.30 (10.51)	136	3.37 (3.00)
Initial visit (viewed lupus PtDA)	48	16.79 (9.51)	153	18.49 (6.42)
Follow-up visit (control)	18	22.45 (15.17)	142	18.49 (6.76)
Non-study patient (control)	37	18.76 (12.81)	290	20.22 (7.57)

*sd* standard deviation, *PtDA* Patient decision aid

Table 3 reports our three estimates of the changes in physician time spent with the patients for in-person visits. When comparing visit lengths for patient visits before which patients viewed the decision aid, to visits by the same patients before which they did not view the decision aid, we find that viewing the decision aid is associated with a mean reduction in visit length of 0.42 min (median reduction of 0.25 min). While these reductions are not statistically significant (*p* = 0.77 for differences in mean, *p* = 0.80 for differences in medians), the results do suggest that viewing the decision aid is not associated with longer visits. Results are more mixed for our estimates made by comparing the unmatched visit times for all study patients. In this comparison, viewing the decision aid is associated with a decrease mean visit length of 1.99 min (*p* = 0.02) but an increased median visit length of 3.88 min (*p* = 0.03). Finally, when comparing the length of visits for all patients who viewed the decision aid to non-study patients who did not, we find that viewing the decision aid is associated with a mean 1.95 min reduction in visit length (*p* = 0.007) and a median reduction of 3 min (*p* = 0.001).

**Table 3:** Estimated Changes in physician time spent with patients (in-person visits)

Physician time (in minutes)	Mean (sd)	Median	25 th percentile	75 th percentile	N
*Estimates based on matched patients only*					
Patients who have viewed the DA before their visit (initial visits)	17.98 (8.55)	16.5	12	23	60
Patients who have not viewed the DA before their visit (follow up)	18.4 (8.48)	16.75	14	20.64	60
Additional time required	− 0.42^a^	− 0.25^b^			
*Estimates based on all patients*					
Patients who have viewed the DA before their visit (initial visits)	18.09 (7.52)	17	14	21	201
Patients who have not viewed the DA before their visit (follow up)	20.08 (8.92)	13.12	15	23.5	160
Additional time required	− 1.99^c^	3.88^d^			
*Estimates based on non-implementation project control patients*					
Patients who have viewed the DA before their visit (initial visits)	18.09 (7.52)	17	14	21	201
Non-implementation project patients	20.03 (8.31)	20	15	24.5	331
Additional time required	− 1.95^e^	− 3^f^			

*sd*, standard deviation

^a ^*p* = 0.77 based on a two-tailed paired t-test of differences in mean visit length

^b ^*p* = 0.80 based on Wilcoxon signed-rank test of medians

^c ^*p* = 0.02 based on a two-tailed, two-sample t-test of differences in mean visit length

^d^ *p* = 0.03 based on a Mann–Whitney test of medians

^e^ *p* = 0.007 based on a two-tailed, two-sample t-test of differences in mean visit length

^f^ *p* = 0.001 based on a Mann–Whitney test of medians

Table 4 describes the additional time sites incur for the non-physician labor required to implement the PtDA. The most time-consuming activity was identifying patients who were eligible to use the PtDA. This activity took and mean time of 41.43 min (median 28.28 min). Interpreting this figure is difficult, however, because sites varied in the process they used to identify eligible patients. Most sites, however, would take time each week, or every other week, to review the schedule of upcoming patients and to identify all scheduled patients eligible to receive the PtDA. Thus, this time figure can be considered a weekly or bi-weekly, rather than a per-patient, time requirement. Presenting the PtDA to patients and explaining how to use it took a mean of 4.12 min and a median of 2 min.

**Table 4:** Time requirements for non-physician implementation activities

Staff time spent (in minutes)	Mean (sd)	Median	25 th percentile	75 th percentile	N
Identification of patients eligible to use the decision aid	41.43 (37.74)	28.28	13.93	63.85	94
Introducing patients to the decision aid and the tablet	4.12 (6.02)	2	2	4	183

Processes for identifying patients eligible for the decision aid differed by sites. However, most sites reviewed eligible patients weekly, for the upcoming week. As a result, the time required to identify eligible patients can be considered a weekly requirement rather than a per-patient requirement

*sd* standard deviation

## Discussion

In this patient education implementation project using a lupus PtDA, we found that the time requirements for implementing the PtDA were minimal. Non-physician clinic staff are responsible for the PtDA activities that take the most time (i.e. identifying patients eligible for the decision aid and introducing the decision aid to patients). It is possible that the most time intensive step in the process could be streamlined. Sites determined the process they used for identifying eligible patients and so there was wide variation in the time required to complete this process step. The middle 50% of recorded observations ranged from 13.93 min required to 63.85 min required. Our findings here are consistent with other studies of decision aid implementation (for uterine fibroids and prostate cancer) which highlight inter-site variation in the time required for PtDA implementation. [15, 16] Like ours, these studies also found that variation in the way sites identify patients eligible for the PtDA is an important driver of variation in the overall time required to implement the PtDA.

Initially we were most concerned with the time requirements that the PtDA created in the patient-physician interaction, since physician time is a clinic’s most expensive resource. However, our results suggest that the PtDA is not likely to increase the length of the physician visit. Of our six different strategies for estimating the change in visit length associated with viewing the PtDA, five demonstrated a reduction in visit length. Despite the high cost of physician time, we were only able to identify one other study that examined the relationship between PtDA use and the length of a physician visit. That study, conducted among patients using a PtDA for prostate cancer, found that the PtDA was associated with a 10 min (or 29%) reduction in visit length, an even larger reduction than the one we observed. [15].

Given that the time required to implement the PtDA appears relatively low, what are the reasons for the observed low-uptake of PtDAs? One potential barrier to adoption is that use of the PtDA is not reimbursable so it would not be expected to increase a clinic’s revenue. However, as value-based reimbursement models become more widespread, benefits like patient satisfaction and cost reductions stemming from less use of other healthcare services (e.g. inpatient hospitalization, emergency department visits) may allow clinics to benefit financially from some of the PtDA’s effects. More broadly, it may be that physicians and clinic staff are so busy that, despite low time-costs and proven benefits for patients, PtDA implementation is drowned out among a sea of competing priorities.

Our findings must be interpreted considering study limitations. First, our findings may not be generalizable to every rheumatology clinic since the data come from only eight rheumatology clinics. However, we Included geographically diverse rheumatology clinics with a mix of academic and private practice clinics to improve generalizability. Another limitation is that our sample size was not large enough to conduct a rigorous investigation of the variation in implementation time across clinics. Finally, our study focused on a single PtDA targeting a single clinical condition. Given the lack of literature describing the time requirements for implementing a PtDA our results are a useful guide, but it is possible that the effects of other PtDAs targeting other conditions may differ from what we have found here. Future studies that can directly observe patient-physician interaction during clinical encounters could provide unique insights into information exchange and verifying time estimates. This would validate findings of the current study.

## Conclusion

Our results, drawn from a detailed time study of over 680 outpatient visits that took place in eight different, geographically diverse rheumatology clinics, suggest that the time burden associated with the implementing and providing lupus PtDA to people with lupus during the regular outpatient visits is low, particularly for physicians. Clinics that make these modest investments in time stand to create a number of important benefits for their patients. Decision-quality in lupus is poor, and many patients decline life-saving immunosuppressive medications, due in part to the lack of recognition of benefits and a fear of harms [17–21]. Patient education programs have been shown to help patients feel comfortable with self-managing day-to-day disease concerns, improving health behaviors, and improving health status [22]. This lupus PtDA (developed with target patient populations, based on their preferences, and updated with most current treatments and evidence to-date) improved patient care by decreasing decisional conflict and facilitating informed choice for immunosuppressive medications use for the treatment of lupus kidney disease [9]. Future research should investigate why PtDA implementation remains low despite these tools’ proven benefits and the modest time required to implement them.

## Data Availability

The quantitative datasets compiled during the study are not publicly available because they would not be useful to others outside the context of the study.
